# Fractional inhibitory concentration of bio-actives from agricultural waste disassembles biofilms and quenches virulence of nosocomial pathogens

**DOI:** 10.1099/jmm.0.001980

**Published:** 2025-03-18

**Authors:** Srividhya Krishnan, Ponnusami Venkatachalam, Saravanan Ramiah Shanmugam, Nithyanand Paramasivam

**Affiliations:** 1 Biofilm Biology Laboratory, School of Chemical and Biotechnology, SASTRA Deemed University, Thanjavur, TN, 613401, India; 2 Biomass, Bioenergy and Bioproducts Laboratory, School of Chemical and Biotechnology, SASTRA Deemed University, Thanjavur, TN, 613401, India; 3 Centre for Bioenergy, School of Chemical and Biotechnology, SASTRA Deemed University, Thanjavur, TN, 613401, India

**Keywords:** biofilms, checkerboard assay, disinfectant, fractional inhibitory concentration, hospital-acquired infections

## Abstract

**Introduction.** The contact surfaces in hospitals serve as reservoirs for pathogens and account for 20–40% of hospital-acquired infections. This resistance is mainly attributed to the biofilm-forming ability of the microbes. These biofilms restrict the entry of the antibiotics to penetrate them, thus giving rise to drug resistance. Hence, there is a renewed interest in formulating an environmentally friendly, non-allergic, quick mode of action, broad-spectrum disinfectant.

**Hypothesis.** We hypothesize that the pure compounds present in the pyrolysis aqueous phase could act as an anti-infective and anti-biofilm agent.

**Aim.** The present work investigates the effectiveness of furfuryl alcohol, 2-methyl-2-cyclopentenone and guaiacol as effective anti-infective agent followed by testing its biofilm eradication potential against the mixed species of multidrug-resistant pathogens such as *Acinetobacter baumannii*, methicillin-resistant *Staphylococcus aureus* and *Candida auris*.

**Methodology.** The MIC and fractional inhibitory concentrations (FIC) of the pure compounds were determined using checkerboard assay for two-compound and three-compound combinations. The biofilm eradication concentration was performed on stainless coupons, followed by RNA isolation and quantitative PCR (qPCR) analysis to elucidate virulence gene downregulation.

**Results.** The individual MICs of furfuryl alcohol, 2-methyl-2-cyclopentenone and guaiacol were found to be 8%, 9% and 2% (v/v), respectively. The two-compound combination FIC index of 0.75 showed partial synergy between the compounds, while the three-compound combination showed an additive effect with a FIC index of 0.87. Further, at ½ FIC (biofilm inhibitory concentration), the compounds showed 52% eradication of preformed biofilms on the hospital contact surfaces (stainless steel). The growth and time-to-kill curve showed that the compounds were not lethal to planktonic cells at BIC. Finally, the qPCR analysis showed a reduction in the expression levels of biofilm and adhesion genes, while the Quorum sensing (QS) genes were affected much more, elucidating a possible eradication mechanism.

**Conclusion.** From this study, we have found a new class of compounds that have potential disinfecting ability. With the current knowledge, the future lead would be to effectively use them in disinfectant formulations.

## Introduction

The ever-rising issue of antimicrobial resistance (AMR) has become a global menace, second only to cancer. Several classes of microbial pathogens now exhibit resistance against the antibiotics that were previously used to fight the infections caused by them. The urge for a new class of antimicrobial agents is the need of the hour. Globally, ~4.95 million deaths have been reported due to AMR in the year 2019. In addition to the deaths, it has an impact on the economy of healthcare sectors, and it is projected that by the year 2030, it will result in an annual Gross domestic product (GDP) loss of around U.S. $304 trillion (WHO, 21 November 2023) [1]. The increase in the usage of antibiotics during the recent Coronavirus disease (COVID-19) pandemic has also given rise to AMR. The mortality rates due to secondary infections were higher post-COVID-19. Infections caused by *Acinetobacter baumannii* and methicillin-resistant *Staphylococcus aureus* (MRSA) are high in hospital settings due to their resistance towards an extended class of antibiotics [2]. It was also reported by the Center for Disease Control and Prevention that the co-infection of *A. baumannii* and MRSA was ~46% in 2020. Apart from bacterial resistance, the World Health Organization (WHO) has also reported *Candida auris* as a critical invasive fungal infection, which urgently requires new antifungal agents for treatment [3].

The most frequent transmission of hospital-acquired infections (HAIs) is through contact or touch surfaces, accounting for ~20–40% of the total HAI rates. It is reported that *A. baumannii* can survive up to 44 °C for 20 days on surfaces and can only be inactivated by moist heat at 70 °C applied for a duration of 30 min [4]. MRSA can sustain for more than 6 weeks on stainless steel surfaces and can only be inactivated by dry heating at 160–170 °C for a period of 1 h [5]. *Candida* infections are the fourth leading nosocomial infections wherein they survive at wide temperature ranges (0–60 °C) and can tolerate a wide pH range of 2.0–8.5. These abilities to withstand extreme temperature ranges, pH tolerance and surface persistence make them more resistant to a wide spectrum of antibiotics. All these attributes are mainly due to their ability to form biofilms. Biofilms are matrices that are produced by the cells to encase themselves. Due to the formation of these surface biofilms, they resist many antimicrobial agents during disinfection measures [6,8]. The disinfection of these surfaces is predominantly performed with ethanol, sodium hypochlorite, UV irradiation, quaternary ammonium salts and some surface cleaning techniques like plasma sterilization. During the pandemic, the U.S. Environmental Protection Agency has listed 545 products (nitrogen-containing groups – list N) that contain quaternary ammonium salts (QASs), alcohols and H_2_O_2_ as active ingredients [9]. Discharge of the chlorine-based chemicals has posed an environmental risk to aquatic ecosystems and sewage systems, while the QASs show adverse effects on respiratory systems and reproductive health [10]. Due to the above-mentioned issues with existing chemical disinfectants, there is a renewed interest in the development of environmentally friendly, non-allergic, quick mode of action, broad-spectrum disinfectants [11].

Pyrolysis is a thermochemical process that breaks down the lignocellulosic biomass into solid (bio-char), liquid (bio-oil aqueous phase) and gas (syngas) fractions [12]. The oil layer of the liquid fraction is used for the fuel applications following catalytic hydrotreatment [13], whereas the aqueous layer is generally regarded as waste and requires further treatment owing to the high chemical oxygen demand and biological oxygen demand before discharging it into the water bodies. The aqueous phase contains oxygenated compounds, aromatics, with various functional moieties along with nitrogen and phosphorus. Hence, direct discharge of the aqueous phase in the water bodies causes eutrophication [14].

In our previous work [14], we have reported that the aqueous phase obtained from the pyrolysis of wheat straw residue had an excellent biofilm eradication property against the pre-formed MRSA biofilms on stainless steel (SS) and polypropylene surfaces which are widely used materials in hospital settings. However, there is a lack of understanding on the inhibitory nature of the major compounds identified from the aqueous phase, i.e. does the inhibition against multidrug-resistant pathogens arise from the individual effect or synergistic effect of these compounds? We envision that addressing this research question would enable us to formulate a disinfectant from agricultural biomass. So, the present work aims to test the individual and combined activity of these pure compounds against mixed-species biofilms of multidrug-resistant pathogens such as * A. baumannii*, MRSA and *C. auris*. We have selected the three compounds, namely furfuryl alcohol, 2-methyl-2-cyclopentenone and guaiacol, based on their higher relative abundance from the GC-MS analysis of the aqueous phase. Fractional inhibitory concentration (FIC), biofilm eradication and downregulation of biofilm and adhesion genes on hospital contact surface (SS coupons) were evaluated to gain a complete understanding of their eradication mechanism.

## Methods

### Procurements of chemicals

The pure compounds of furfuryl alcohol, 3-methyl-2-cyclopentenone and guaiacol were purchased from Sigma-Aldrich (purity 99.0%). Guaiacol was dissolved in 50% ethanol to a stock concentration of 40% (v/v) and then diluted using 1X PBS, while the other two compounds were dissolved using water. Soybean casein digest (SCD) broth, yeast peptone broth (YPD) and Roswell Park Memorial Institute (RPMI) were purchased from HiMedia, India.

### Microbial strains and culture conditions

Clinical isolates of *A. baumannii* (AB2) (GenBank accession no. MF443128), MRSA (GSA-44) and *C. auris* (GenBank accession ID MK108049) strains were used in the study. MRSA and *A. baumannii* were cultured in SCD and *C. auris* was cultured in a YPD medium. All three cells were adjusted to a cell density of 10^7^ c.f.u. ml^−1^ separately, and then 16.6 µl from each cell suspension were added, so that the total volume of cells is 50 µl wherein the final cell density was 3×10^7^ c.f.u. ml^−1^. For the co-culturing of bacterial and fungal cells, RPMI medium is used.

### Determination of MIC

The minimum concentration required to inhibit the growth of the test strains was tested using the MIC assay [15]. Briefly, in a 96-well plate, 100 µl RPMI media, 50 µl of cells and 50 µl of compound on varying dilutions were added and kept for incubation at 37 °C for 24 h. Following incubation, 30 µl of MTT (3-(4,5-dimethylthiazol-2-yl)-2,5-diphenyltetrazolium bromide) dye was added and incubated in the dark for 30 min. The results were visualized and recorded.

### Determination of FIC of test compounds in two-compound combinations

A checkerboard assay was performed to check the synergistic activity of the three test compounds in three different combinations. Combination 1 comprised furfuryl alcohol and 3-methyl-2-cyclopentenone, combination 2 comprised 3-methyl-2-cyclopentenone and guaiacol and combination 3 comprised guaiacol and furfuryl alcohol. The compounds were prepared in the range of eight times their respective MIC [16,17]. To a 96-well plate, 100 µl of RPMI media, 50 µl of cells and 50 µl of varying concentrations of the compounds were added according to the above-mentioned combinations. The plates were incubated at 37 °C for 24 h. After incubation, the plates were screened for their fractional inhibitory concentrations by adding 30 µl concentration MTT. Equation 1 gives the FIC index of two compound combinations.

(1) $$FIC\left(oftwocompounds\right)=\frac{MIC\left(1incombination\right)}{MIC1}+\frac{MIC\left(2incombination\right)}{MIC2}$$

### Determination of FIC of test compounds in three-compound combinations

To determine the FIC of three compounds in combination, the FIC of two combinations is fixed as MIC, and the third compound is varied accordingly [17]. As mentioned above, 100 µl of RPMI media and 50 µl of cells were added to a 96-well plate. Fifty microlitres of varying concentrations of compounds were added and incubated at 37 °C for 24 h. Following the incubation, 30 µl concentration MTT was added and visualized. Equation 2 was used to calculate the FIC index of the three-compound combination.

(2) $$FIC\left(ofthreecompounds\right)=\frac{MIC(\left(1,2\right)incombination)}{MIC\left(1,2\right)alone}+\frac{MIC(3incombination)}{MIC\left(3\right)alone}$$

FIC<0.5 is synergy, 0.5<FIC≤0.75 is partially synergy, 0.75<FIC≤1 is additive, FIC>1 is indifferent and FIC>4 is antagonistic.

### Determination of biofilm eradication ability at its FIC

To check for the matured biofilm disrupting ability of the three compounds in combination, a biofilm eradication assay was performed. In a 24-well plate, 1 ml of media with 100 µl cells was incubated for 24 h at 37 °C under static conditions [15]. After 24 h, the spent media was removed, and the fresh media was supplemented along with 50 µl of the three compounds at their 1/2^nd^, 1/4^th^ and 1/8^th^ of FIC and incubated for another 24 h. Following incubation, the spent media was removed, and the biofilms were stained with 0.4% crystal violet (CV). Excess stain was washed with water, wells were solubilized with 70% ethanol and the absorbance was recorded using an ELISA plate reader (Tulip, Lisaquant, TS). The wells without compounds served as controls. The biofilm eradication was calculated using Equation 3.

(3) $$Percentagebiofilmeradication(\%)=\frac{ControlOD-SampleOD}{ControlOD}*100$$

### Determination of planktonic cell growth by growth curve analysis

To check if the compounds inhibit cell growth, a growth curve assay was performed. Briefly, 1% (v/v) of the overnight cultures was inoculated into RPMI media. The compounds at concentrations of 1/2 BIC and FIC were added and incubated at 37 °C at 150 r.p.m. The tubes with only cells served as controls. The cell absorbance was recorded for every 2 h at 600 nm [18].

### Determination of viable cell count by time-kill analysis

To support that the planktonic cells are alive, a time-kill assay was performed. The mixed cell suspensions without treatment, FIC treated and BIC treated were prepared. The samples were collected at 2-h time intervals, serially diluted and spread plated in the SCD and YPD agar plates and incubated at 37 °C for 24 h. The countable colonies were recorded, and log c.f.u. was calculated accordingly [18].

### Visualization of biofilm eradication through light microscopy

The Minimum Biofilm Eradication concentration (MBEC) assay was performed on a 1×1 cm glass slide. The slides were stained using 0.4% CV and air dried. The CV binds to the Exopolysaccharide (EPS) produced by the biofilms. The control and treated slides were then visualized under the light, and images were captured using Nikon Eclipse Ti 100, Japan digital camera.

### Visualization of the cell viability using live/dead staining

The viable but non-culturable cells were visualized using a live/dead staining using SYTO9 and Propidium Iodide (PI) dyes and observed under the fluorescence microscope (Model-Eclipse Ts2, Serial No. 245859) [19].

### Effect of biofilm eradication on contact surface and molecular level downregulation using the qPCR analysis

To elucidate a mechanistic view of the action of these compounds, the major genes responsible for biofilm formation, quorum sensing and adhesion were studied through the quantitative PCR (qPCR) analysis. On the SS (Grade 316) coupons, the MBEC assay was performed on a 6-well plate. The coupons with only cells served as the control. RNA was isolated from scrapping the biofilms present in the SS coupons using 1X PBS as reported elsewhere [19]. First, the biofilm cells were centrifuged followed by (1:1) phenol/chloroform extraction. To the obtained aqueous phase, lysis buffer was added, and the RNA was precipitated using isopropanol. The obtained pellet was washed with ice-cold 70% ethanol and centrifuged at 5000 r.p.m. for 5 min. The RNA pellet was air dried and suspended in 25 µl RNase-free water and stored at −80 °C. The RNA was converted to cDNA using the High-Capacity Reverse Transcription Kit 260 (Applied Biosystem™, USA). The gene expression of the virulence genes as listed in the Table SA4 was quantified using real-time StepOnePlus thermal cycler 262 (AB17500, Applied Biosystems™, USA) using SYBR green PCR mix.

### Visualization of biofilm eradication on SS surface through scanning electron micrographs

The biofilm eradication was performed on SS coupons, the Scanning Electron Microscope (SEM) samples were prepared as per [14] by fixing with glutaraldehyde and dehydration with ethanol. The samples were gold sputtered and visualized (VEGA, TESCAN).

### Statistical analysis

All the studies were carried out in triplicates. The results are expressed as mean±sd, and data were analysed using ANOVA. The significant difference between the control group and treated groups was indicated using asterisks (*) and *P*<0.05. Graphs were plotted using GraphPad Prism version 5.

## Results

### Individual compounds possess microbicidal activity against mixed microbial pathogens

The MIC determines the least concentration of the compound needed to kill the cells. The formation of the formazan product by the live cells is visualized by the purple colour. The MTT assay shows a MIC of 8%, 9% and 2% (v/v) for furfuryl alcohol, 3-methyl,2-cyclopentenone and guaiacol, respectively, against the mixed microbial community of *A. baumannii*, MRSA and * C. auris* (Fig. SA1 and Table SA3, available in the online Supplementary Material).

### Evaluation of FIC of two compounds against mixed microbial pathogens

**Fig. 1.**
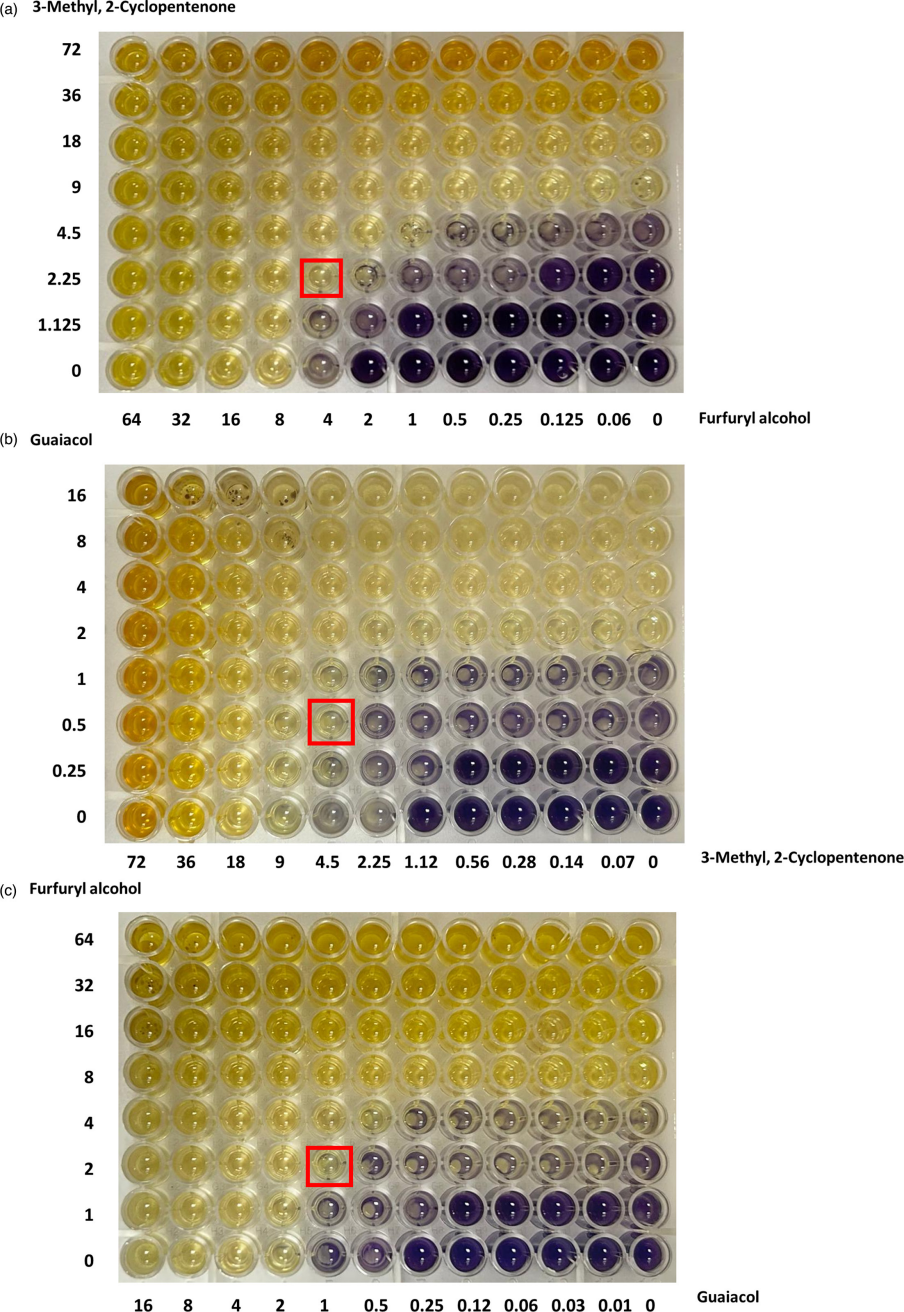
The FIC of two-compound combinations. (a) Furfuryl alcohol and 3-methyl,2-cyclopentenone, (b) 3-methyl,2-cyclopentenone and guaiacol and (c) guaiacol and furfuryl alcohol.

The two-compound combinations were assessed using the checkerboard assay. The use of two compounds in combination can possess an additive, synergistic or antagonistic effect depending on their mode of action. In our case, all the three combinations showed an FIC index of 0.75 inferring they are partially synergistic to each other (Fig. 1a–c). The FIC of the combinations was calculated using Equation 1 and is tabulated in Table 1.

**Table 1.** FICs of two compounds at three different combinations against mixed microbial strains FIC 1 (furfuryl alcohol, 3-methyl,2-cyclopentenone)=4/8 + 2.25/9=0.75 (partial synergy). FIC 2 (3-methyl,2-cyclopentenone, guaiacol)=4.5/9 + 0.5/2=0.75 (partial synergy). FIC 3 (guaiacol, furfuryl alcohol)=1/2 + 2/8=0.75 (partial synergy).

Compound	FIC index	Inference
**Furfuryl alcohol, 3-methyl,2-cyclopentenone**	0.75	Partial synergetic
**3-Methyl,2-cyclopentenone, guaiacol**	0.75	Partial synergetic
**Guaiacol, furfuryl alcohol**	0.75	Partial synergetic

### Evaluation of FIC of three compounds against mixed microbial pathogens

**Fig. 2.**
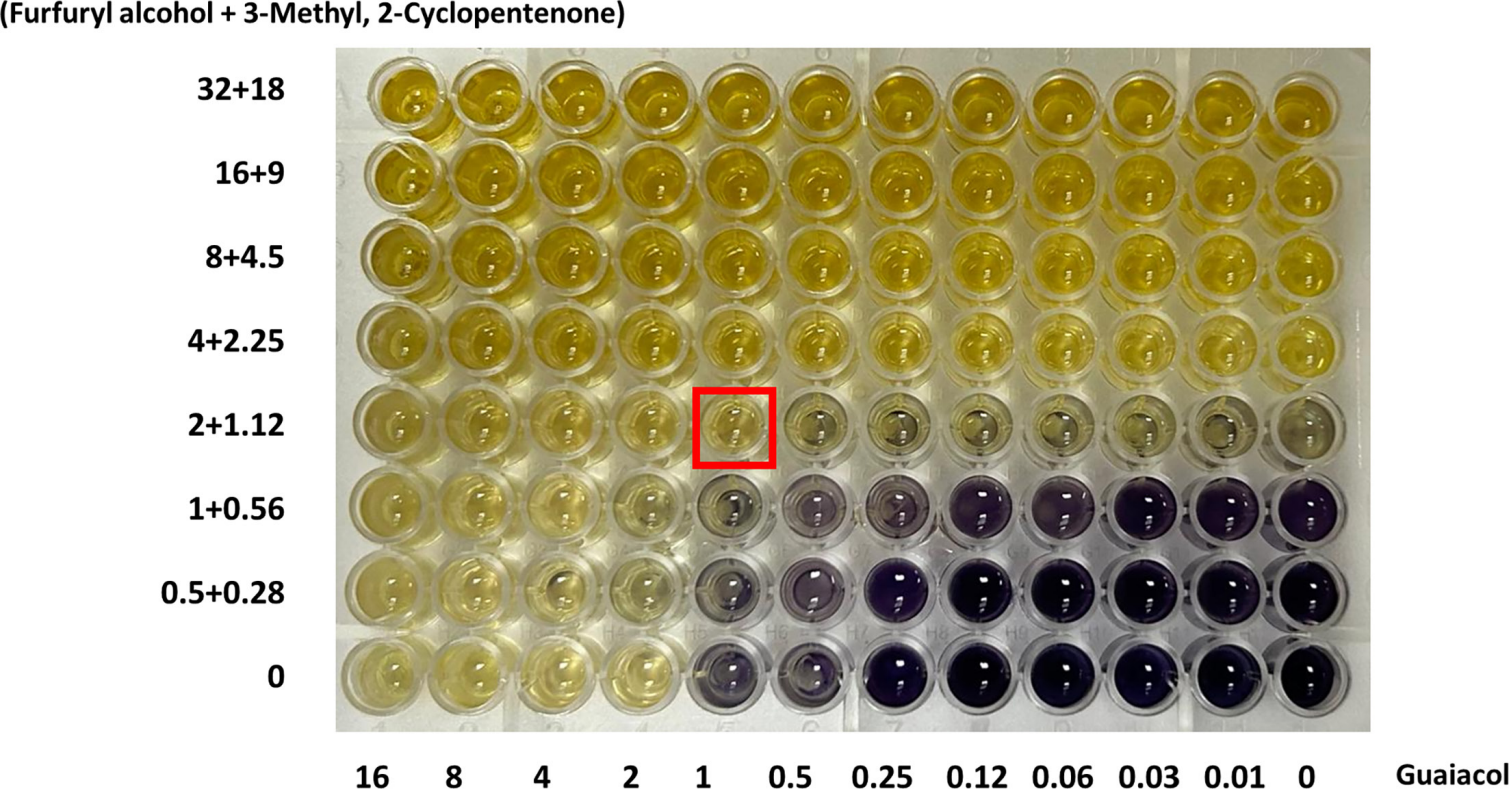
The FIC of three compounds where 3-methyl,2-cyclopentenone and furfuryl alcohol were considered as single compounds and guaiacol being the second compound.

The FIC of all the three compounds was assessed by taking 3-methyl,2-cyclopentenone and furfuryl alcohol together against guaiacol (Fig. 2). The three-combination FIC was calculated using Equation 2. The combination showed an additive effect with a FIC index of 0.87 (Table 2).

**Table 2.** FICs of three compounds against the mixed species FIC (furfuryl alcohol + 3-methyl,2-cyclopentenone) / guaiacol=2/8 + 1.12/9 + 1/2. FIC index (combined)=0.87.

Compound combination	FIC index (combined)	Inference
**Furfuryl alcohol+3-methyl,2-cyclopentenone/guaiacol**	0.87	Additive

The additive effect was observed in the three compound FIC, indicating that all three compounds are responsible for the activity and it is not the individual or synergistic effect even though they are present as a mixture.

### FIC concentration of compounds eradicated mixed species biofilms

**Fig. 3.**
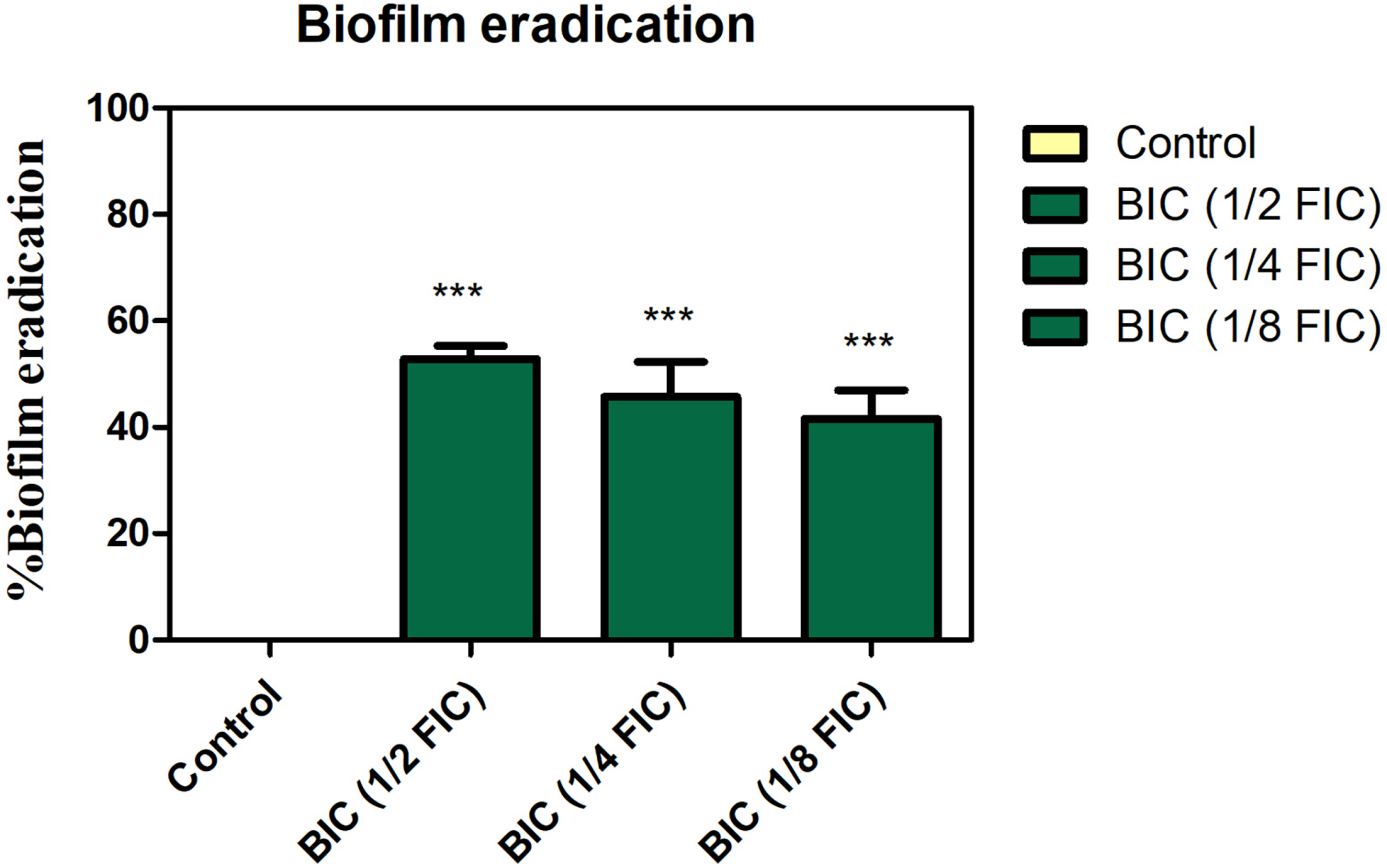
Biofilm eradication ability of the pure compounds at 1/2 FIC, 1/4 FIC and 1/8 FIC.

The ability of the three compounds against the eradication of pre-formed biofilms was evaluated at its 1/2^nd^, 1/4^th^ and 1/8^th^ FIC concentrations. It was shown to eradicate 53% of the mixed species biofilms at 1/2^nd^ FIC concentration (Fig. 3). This concentration was considered as its biofilm inhibitory concentration (BIC) for further studies.

### BIC concentration was non-lethal against planktonic cells

**Fig. 4.**
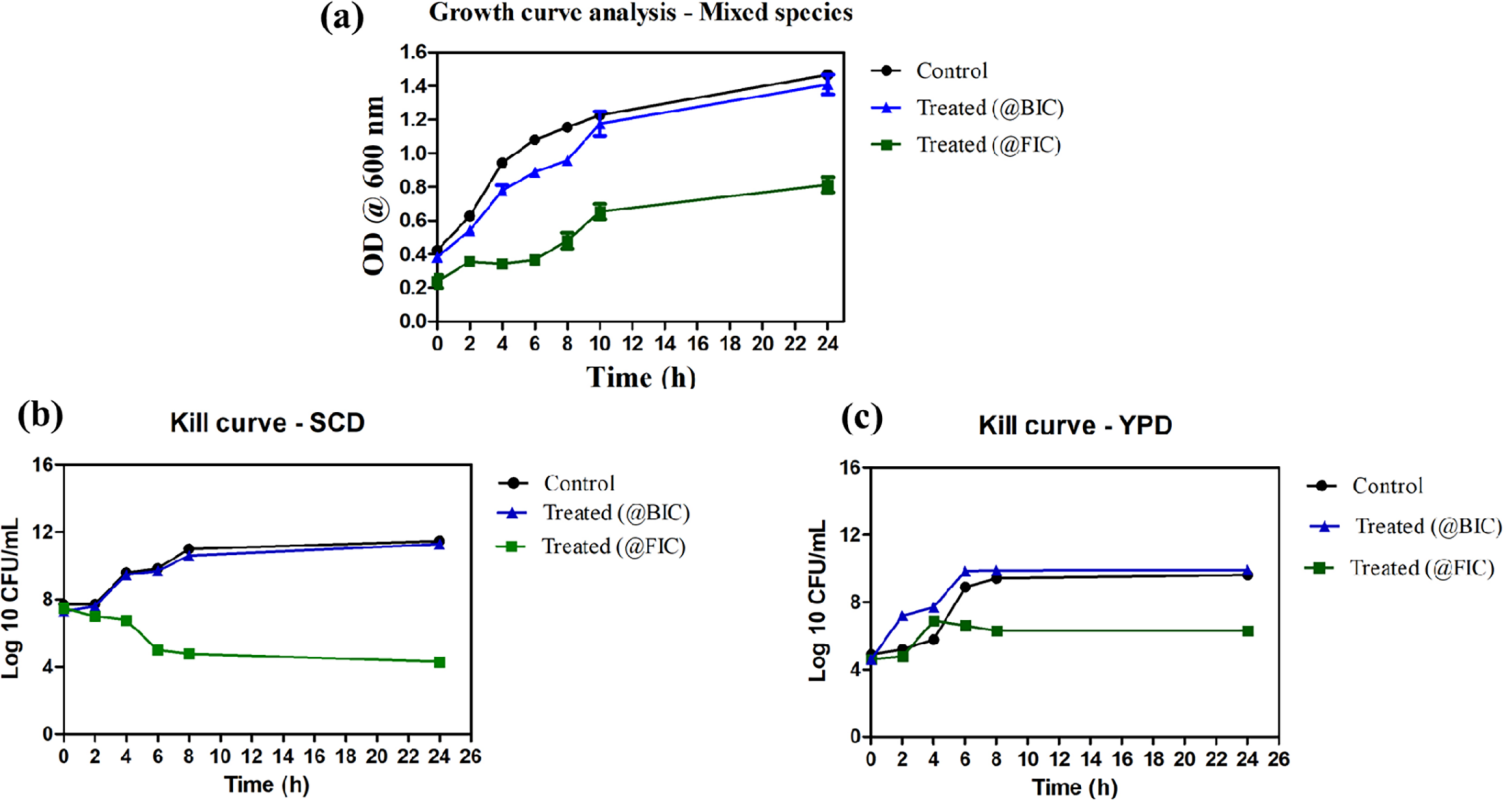
(a) The growth pattern of mixed species in RPMI medium upon treatment at its BIC and FIC and control. Time-kill pattern on (b) SCD agar and (c) YPD agar showing log c.f.u. reduction at FIC, BIC and control is shown.

The microbicidal activity of the FIC and BIC concentrations was assessed against mixed microbial strains. Cells without treatment served as control. It was observed that the FIC concentration showed a decline in the cell growth, indicating that it was lethal to the microbial cells. On the other hand, the BIC concentration did not show any lethal effect against the cells, as the cells treated with it showed a growth pattern similar to that of the control (Fig. 4a).

A time-kill curve pattern was obtained by enumerating the colonies and calculating their c.f.u. per millilitre. The time-kill plot shows that the BIC was not lethal to the planktonic cells, while at FIC, the compounds exhibited killing effects. Since the mixed species contain *A. baumannii*, MRSA and *C. auris*, the samples were spotted on SCD agar and YPD agar plates to enumerate bacterial and fungal cells, respectively. The cells were reduced by 4.3 log c.f.u. ml^−1^ (in SCD) and 6.3 log c.f.u. ml^−1^ (in YPD) at its FIC (Fig. 4b, c), whereas the BIC showed a similar growth pattern as the control, which further indicates that BIC does not kill the bacterial and fungal cells. From the growth curve and kill graph, we can correlate and infer that the control and BIC-treated cell absorbance were increasing in the growth curve (Fig. 4a) with an increase in log c.f.u. per millilitre from 2 h itself (Fig. 4b, c). Meanwhile, there is reduced cell growth in the case of FIC treated with reducing log c.f.u. per millilitre.

### Biofilm eradication of mixed species visualized using light microscopy

MBEC assay performed with BIC concentration on glass slides were visualized after CV staining under a light microscope. CV binds to the EPS, and as a result, the intensity of CV aggregation was high in the control sample and lesser in the case of the BIC-treated sample, as shown in Figure SA2.

### Visualization of viable but non-culturable cells through fluorescence microscopy

**Fig. 5.**
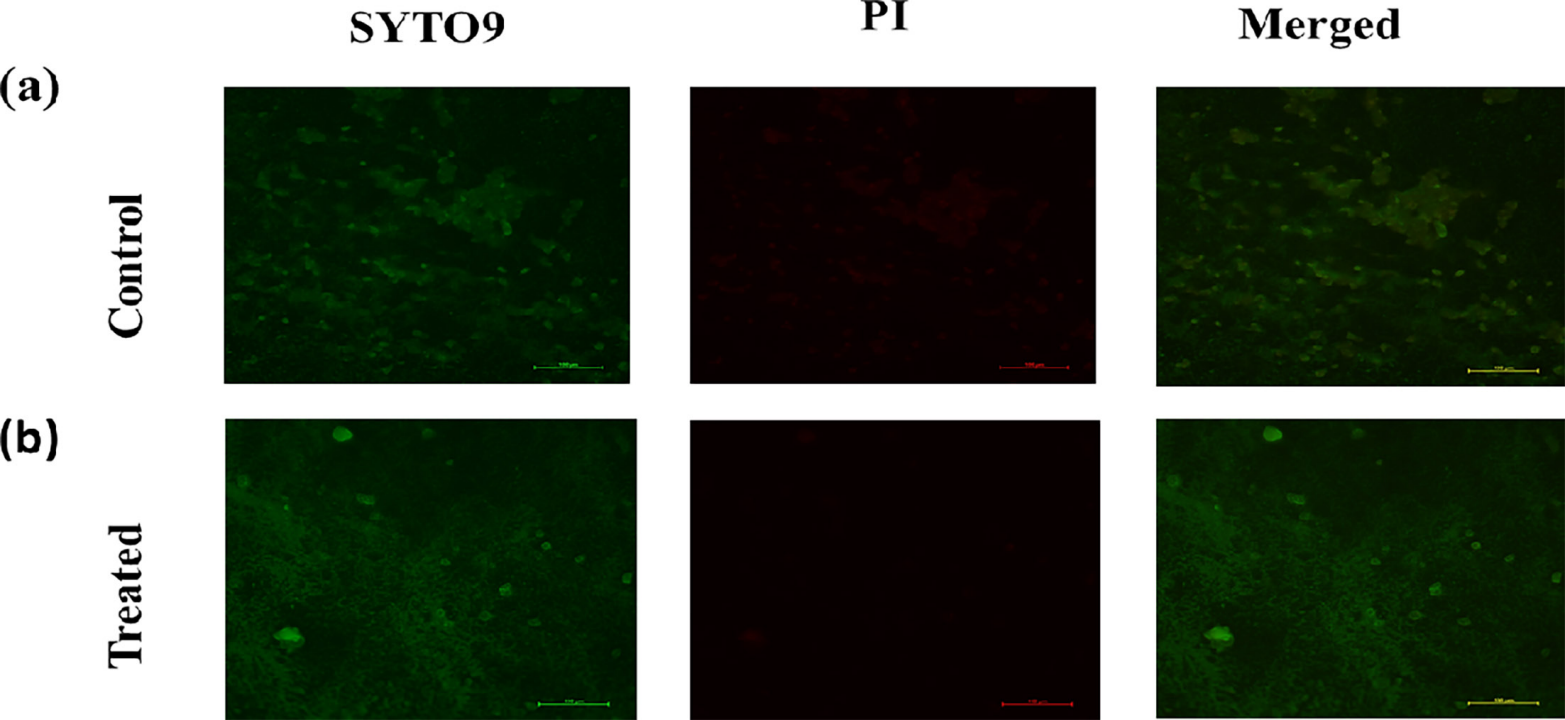
Fluorescence microscopy image showing live and dead cells staining of mixed species. (a) Control slide and (b) BIC-treated slide showing green (SYTO9), red (PI) and merged fluorescence.

Fluorescence microscopic images show us the metabolically inactive cells that are not culturable. The SYTO9 dye binds to the DNA of live cells and emits green fluorescence, while PI can only enter and bind to the DNA of cells with a compromised membrane, emitting red fluorescence, indicating the presence of dead cells. In the case of control, the cells were metabolically alive emitting green fluorescence, while in case of BIC treated, the cell morphology was changed, but the green intensity tells that the planktonic cells were still alive. The green fluorescent intensity of the BIC-treated sample was less than the control due to a reduction in biofilm cells (Fig. 5). The treated samples did not show any red fluorescence indicating that the cells were not affected much.

### Mixed species biofilm eradication on an SS surface downregulates the virulence genes

**Fig. 6.**
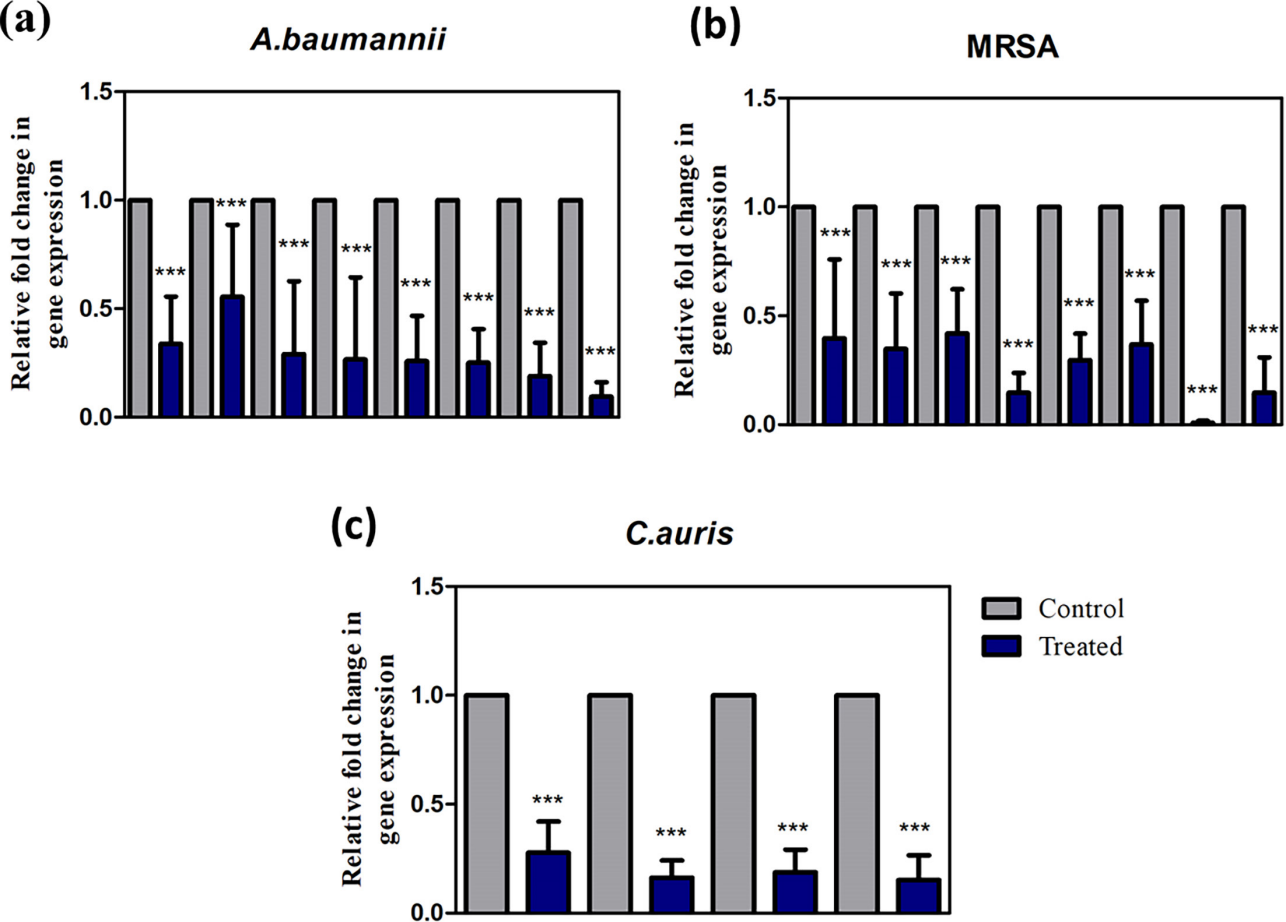
The qPCR analysis of virulence gene expression of (a) MRSA, (b) *A. baumannii* and (c) *C. auris* in mixed species biofilms developed on control and treated SS surfaces.

The downregulation of virulence genes was observed in MRSA, *A. baumannii* and *C. auris*. The genes used, along with their function and primer sequences, are listed in Tables SA4 and SA5. In the case of MRSA, the EPS adhesion genes (*ica*A, *ica*D) and staphyloxanthin genes (*crt*M and *crt*N) were downregulated to 0.36-fold, while the expression of fibronectin binding protein (*fnb*B) and autoinducer (*ssp*B) genes were decreased to 0.14- and 0.14-fold, respectively. The effect on *agr*AC, which modulates the Quorum sensing (QS) virulence, was greatly affected (Fig. 6a).

In *A. baumannii*, genes related to surface motility (*aba*I and *aba*R) were reduced to 0.34- and 0.55-fold, respectively. Downregulation in genes related to abiotic surface attachment (*bfm*R, *bfm*S) and initial attachment (*csu*A/B, *csu*E) was observed at the range of 0.25–0.29-fold. Genes responsible for the structural integrity of the biofilms (*pga*B) were downregulated by 0.19-fold, while the fibronectin binding gene (*omp*A) was downregulated to 0.10-fold as observed in MRSA (Fig. 6b). In the case of *C. auris*, fold reduction was observed in the *erg* gene which is responsible for azole resistance, while the other genes responsible for biofilm formation (*efg*1, *hgc*) and efflux (*cdr*) were reduced to 0.18- and 0.15-folds, respectively (Fig. 6c).

### Visualization of biofilm eradication on SS surfaces

**Fig. 7.**
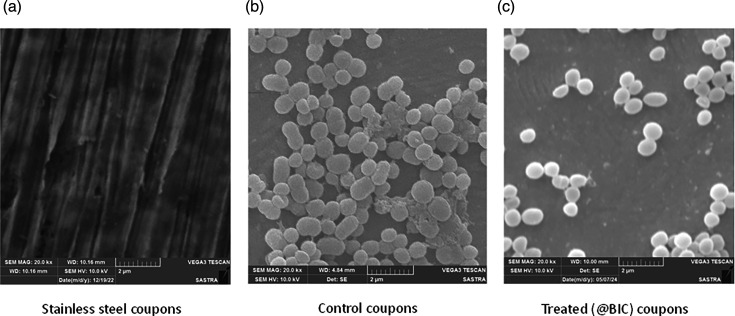
Scanning electron microscopy images of mixed species (a) plain SS coupon, (b) control SS coupon and (c) BIC-treated SS coupon.

From the SEM images, it can be clearly seen that the biofilms were disrupted by the compounds. The control and treated photographs show the clear difference between the matured biofilms in the control sample and dismantled biofilm architecture in the treated sample (Fig. 7). A clear EPS reduction can be observed in the case of the BIC-treated SS coupons (Fig. 7c).

## Discussion

Healthcare facilities serve as a vector for the transmission of micro-organisms causing HAIs. Apart from infected patients, transmission takes place from frequently touched surfaces like door handles and switches, which are known as ‘high-touch surfaces’ [20]. Disinfection is the preliminary way of controlling any infection on inanimate objects. Currently, the majority of healthcare surface settings use neutral detergents, hypochlorite or ethanol as disinfecting agents as suggested by the European Centre for Disease Prevention and Control [21]. One of the major reasons for these microbes to gain drug resistance is through their biofilm-forming ability. It was reported that ~65% of HAIs were caused by biofilm formation [22]. The technically important stages of the biofilm include the initial attachment, microcolony formation followed by maturation and finally dispersion. It is reported that the biofilms increase the resistance of conventional antibiotics by 1000-fold [23]. Various anti-biofilm molecules have been identified from natural sources, synthetic compounds and lantibiotics. The rise in demand for novel strategies is mainly focused on two approaches: (i) development of biofilm inhibitors or (ii) surface modification [24]. In clinical settings, the biofilms are polymicrobial. In view of that, the present study has been fully performed on mixed microbial species conditions. So, an effective anti-biofilm agent should be able to penetrate them to provide the necessary mode of action. The inter- and intra-kingdom interactions must be elucidated in order to provide a complete understanding of the mechanisms, as they promote the virulence and recalcitrance of biofilms [25]. Studies have shown the use of plant-based anti-biofilm agents like flavonoids, organic acids and phenolics to target MRSA. Santiago *et al.* [26] showed a decrease in cell surface attachment of the biofilms when treated with extracts of the F-10 fraction of *Duabanga grandiflora* extract. The gallic and ellagic acids present in the extract of *Cochlospermum regium* inhibited the biofilm by decreasing the polysaccharide secretion in MRSA and Methicillin sensitive *Staphylococcus aures* (MSSA) [27]. The alteration in biofilm structure and staphyloxanthin production was reported using Pulverulentone A, 8-desmethyl eucalyptin and eucalyptin isolated from methanol extracts of *Callistemon citrinus* [28,29]. Previous reports have shown that hexane and dichloromethane extracts of leaves of *Allium stipitatum* and major compounds of *Syagrus coronata* (octanoic acid, dodecanoic acid, decanoic acid and *γ*-eudesmol) cause disintegration of matured biofilms by penetrating deep inside the matrix [30,31]. Alibi *et al.* [32] showed biofilm inhibition against *A. baumannii* using *Thymus vulgaris*, *Cinnamomum verum* and *Eugenia caryophyllata* of 88%, 97% and 91%, respectively. Methanolic extracts of *Carum copticum* showed disruption in biofilm structure reported to have thymol (40%), *ϒ*-terpinene (36%) and p-cymene (21%) as major compounds against six pathogens, namely *Bacillus cereus*, *S. aureus*, *Pseudomonas aeruginosa*, *E. coli*, *A. baumannii* and *Klebsiella pneumonia* [33]. The DCM extract of the bulbs of *Allium stipitatum* showed the reduction in biofilm viability and structure dismantling of MRSA and *A. baumannii* biofilms [34]. All these methods have used the direct plant extracts obtained through solvent extraction processes, which are basically the secondary metabolites produced by the plants. But in our work, we have proposed a thermochemical conversion process known as pyrolysis as an effective way of utilizing the biomass. In this technique, the biomass is subjected to higher temperatures, which on degradation produce volatile matters that are condensed as an aqueous phase.

In our previous work [14], we showed that the wheat straw aqueous phase showed a potential anti-infective effect in disrupting the biofilms formed on SS. Through docking studies, we have also predicted the major compounds, namely furfuryl alcohol, 3-methyl,2-cyclopentenone and guaiacol obtained through GC-MS analysis as responsible compounds for this activity. So, the present work focused on testing those individual compounds to elucidate whether these compounds possess synergistic or additive effects. In this study, the individual compounds tested showed MIC at 8%, 9% and 2% (v/v) for furfuryl alcohol, 3-methyl,2-cyclopentenone and guaiacol, respectively, against the mixed species of pathogens. The two-compound combination an FIC showed a FIC index of 0.75, implying partial synergy between the compound combinations. Followed by the FIC of three-compound combinations showed an additive effect with an FIC index of 0.87, confirming that the anti-infective activity was due to the combinational effect and not due to a synergistic effect. Guaiacol, also known as methyl catechol, is the major product obtained from wood distillation. It has widely been used as an antiseptic and local anaesthetic. Studies have shown its antibacterial and antifungal activity. It was also shown to inhibit the growth of *Fusarium graminearum*. At 1.838 mM (2.05×10^−4^%), guaiacol has inhibited conidial production and germination by disrupting Ca^2+^ channel with decreased levels of catalase, peroxidase and superoxide dismutase acting as an effective antioxidant [35]. Further, guaiacol oligomers synthesized by polymerization reaction showed excellent antioxidant and antimicrobial activity by reducing the bacterial load of *E. coli* and *S. aureus* [36] [37]. Chai *et al.* have reported furan compounds as novel tyrosinase inhibitors and antimicrobial agents with MIC of furfuryl alcohol to be 0.115 µM against *Bacillus subtilis* and *Staphylococcus bacteria*. A study by Goa *et al.* [38] has reported the by-product of pyrolysis of wheat straw condensates as an effective fungicide. They showed an antifungal activity against *F. graminearum* at a concentration of 3.1 µl ml^−1^, showing a higher potential than the synthetic fungicide. A study by Amin *et al.* [39] has reported cyclopentenone derivatives as an effective antifungal agent against *Aspergillus sydowii*.

Since biofilms were one of the potential targets, we extended our study to find the biofilm eradication ability of these compounds. At 1/2^nd^ FIC, they showed ~53% biofilm eradication against the mixed species and hence were chosen as BIC. The growth curve showed that there is no reduction in planktonic cell growth in control and BIC-treated samples, which is supported by the kill curve plots, which show an increase in log c.f.u. in control and BIC-treated samples. The non-culturable, viable cells were also visualized using live/dead fluorescence microscopy. The EPS reduction was also confirmed through SEM images.

The initial attachment stages are governed by the surface-associated proteins and biofilm-associated proteins (*bap*, *ompA*, *fnaA* and *fnbB*). Some species directly anchor to the matrix and mediate colonization. *S. aureus* can directly form larger aggregates (*icaA*, *icaD*, *sspB*, *csu* and PNAG). To understand this mechanism, we tested it on the high-touch surfaces of hospitals such as SS surfaces. The eradication was performed on SS coupons. To understand the genetic level changes, the qPCR analysis was performed by extracting the RNA from the SS coupons. It can be understood from the downregulation pattern that the genes responsible for surface motility (*aba*I and *abaR*) and EPS adhesion (*ica*) were significantly downregulated followed by the abiotic surface attachment (*bfmR* and *bfmS*) and biofilm formation genes (*efg*), while the QS genes (*agrAC*) and autoinducing peptide (*ssp*) were affected greatly. This array pattern unfolds the possible biofilm eradication mechanism.

The present study shows that it is the additive effect of the compounds (furfuryl alcohol, 3-methyl,2-cyclopentenone and guaiacol) present in the aqueous phase that is responsible for this anti-infective property and not the individual compound. The screened compounds show both anti-biofilm property at their BIC and anti-infective property at their FIC. The compounds were efficient in eradicating the pre-formed biofilms on high-contact SS surfaces. The qPCR analysis showed that this activity is due to the downregulation in biofilm genes, while the QS system was most affected.

## Conclusion

The study highlights that an additive effect of the compounds present in the aqueous phase is only responsible for the observed property and it is not the effect of a single compound. To conclude, the leads from the present study would be to investigate the formulation aspects of the major compounds present in the aqueous phase as a disinfectant coating to be used on inanimate hospital materials and surfaces that act as reservoirs of multi-drug resistant (MDR) pathogens.

## supplementary material

10.1099/jmm.0.001980 Uncited Fig. S1.

## Abbreviations

AMR: antimicrobial resistance
BIC: biofilm inhibitory concentration
CV: crystal violet
EPS: exopolysaccharide
FIC: fractional inhibitory concentrations
GDP: gross domestic product
HAIs: hospital-acquired infections
MBEC: minimum biofilm eradication concentration
MDR: multi-drug resistant
MRSA: methicillin-resistant *Staphylococcus aureus*
MSSA: methicillin sensitive *Staphylococcus aures*
MTT: 3-(4,5-dimethylthiazol-2-yl)-2,5-diphenyltetrazolium bromide
PI: propidium iodide
QASs: quaternary ammonium salts
qPCR: quantitative PCR
QS: quorum sensing
RPMI: Roswell Park Memorial Institute
SCD: soybean casein digest
SEM: scanning electron microscope
SS: stainless steel
WHO: World Health Organization
YPD: yeast peptone broth
