# Supplementary material for: Fractional inhibitory concentration of bio-actives from agricultural waste disassembles biofilms and quenches virulence of nosocomial pathogens
Source: J Med Microbiol. 2025 Mar 18;74(3):001980. doi: 10.1099/jmm.0.001980 (PMC11920071; doi:10.1099/jmm.0.001980)
Supplement: Uncited Fig. S1. [file jmm-74-01980-s001.pdf]

Supplementary material

Manuscript title: **“**Fractional Inhibitory Concentration of bio-actives from agricultural waste disassembles biofilms and quenches virulence of nosocomial pathogens” by **Srividhya et al.,**

Manuscript number: JMM-D-24-00513.R1

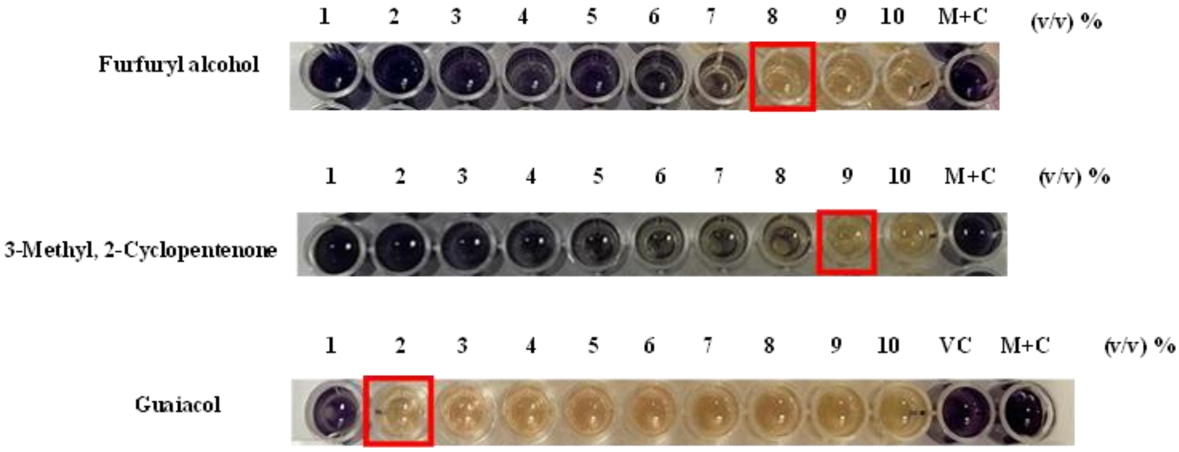

Figure A1. Minimum inhibitory concentration (MIC) of compounds Furfuryl alcohol, 3-Methyl, 2-Cyclopentenone and Guaiacol at 8%, 9% and 2% respectively highlighted in red box.

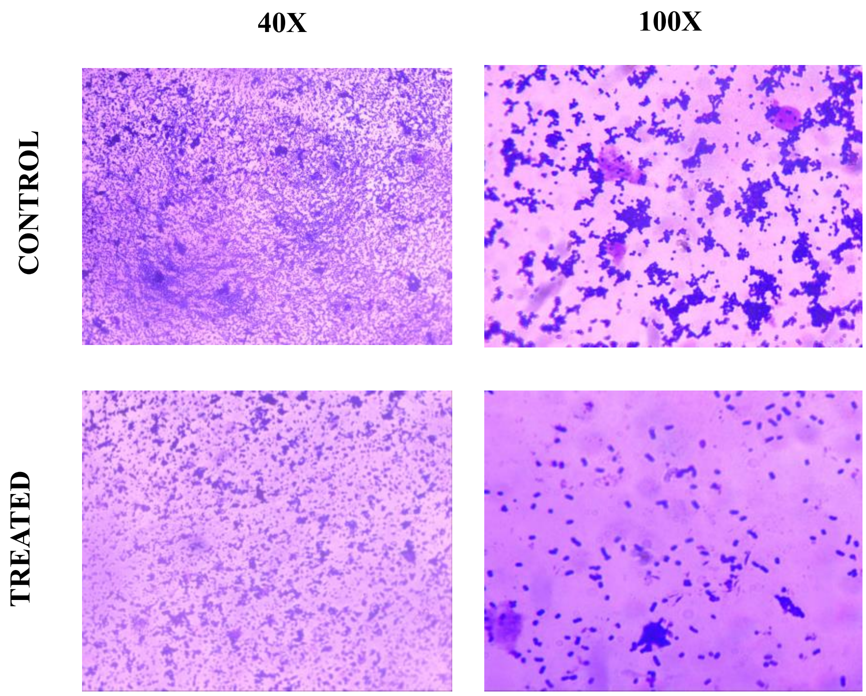

Figure A2. Light microscopic visualisation of biofilm eradication observed in the control and BIC treated sample at 40X and 100X magnifications

Table A3. The Minimum inhibitory concentration (MIC) of compounds Furfuryl alcohol, 3-Methyl, 2-Cyclopentenone and Guaiacol at 8%, 9% and 2% against the mixed species.

COMPOUNDS	MIC (%)
Furfuryl alcohol	8
3-Methyl, 2-Cyclopentenone	9
Guaiacol	2

Table A4. Primer's list of MRSA, *A. baumannii* and *C. auris* along with along with their role

S.No	Gene	Sequence (5'-3')	Role
1	*agrAC* (F)	CTGATAATCCTTATGAGGTGC	QS virulence factors
	agrAC (R)	CGATGCATAGCAGTGTTC	
2	*icaA*(F)	ACACTTGCTGGCGCAGTCAA	EPS adhesion
	icaA(R)	TCTGGAACCAACATCCAACA	
3	*icaD*(F)	ATGGTCAAGCCCAGACAGAG	EPS adhesion
	icaD(R)	AGTATTTTCAATGTTTAAAGCA	
4	*crtM*(F)	ATCCAGAACCACCCGTTTTT	Staphyloxanthin inhibition
	crtM(R)	GCGATGAAGGTATTGGCATT	
5	*crtN*(F)	GATGAAGCTTTGACGCAACA	Staphyloxanthin inhibition
	crtN(R)	TTCGCATGATACGTTTGCTC	
6	*fnbA*(F)	ATCAGCAGATGTAGCGGAAG	Fibronectin binding gene
	fnbA(R)	TTTAGTACCGCTCGTTGTCC	
7	*fnbB*(F)	AAGAAGCACCGAAAACTGTG	Fibronectin binding gene
	fnbB(R)	TCTCTGCAACTGCTGTAACG	
8	*sspB*(F)	CCAGCAAATTGTTGTTGTGCTAG	Autoinducer signalling molecule
	sspB(R)	AAGCCAAAGCCGATTCACACTC	
9	*csu A/B* (F)	CAGCAGCAACAGGTGGCAATA	Initial attachment
	csu A/B (R)	AAGGTTTGTACGTGCAGCATCA	
10	*csuE*(F)	GCTTGGCTTTAGCAAACATGACC	Initial attachment
	csuE(R)	ATTGCCATCAGGCCCGCTA	
11	*bfmS*(F)	ACCGCCCGTAATCCGAAC	Abiotic surface
	bfmS(R)	TGAACTTATTCCACCGCCTTTA	
12	*bfmR*(F)	GTTTAACCGTTTGTCGTG	Abiotic surface
	bfmR(R)	GTGGTTGAACTGGTTTCG	
13	*pgaB*(F)	AAGAAAATGCCTGTGCCGACCA	Structural integrity
	pgaB(R)	GCGAGACCTGCAAAGGGCTGAT	
14	*ompA*(F)	CGCTTCTGCTGGTGCTGAAT	Fibronectin binding gene
	ompA(R)	CGTGCAGTAGCGTTAGGGTA	
15	*abaR*(F)	ATGGAAAGTTGGCAAGAG	Surface motility
	abaR(R)	CTACAAAAGCCCTAGCATTAC	
16	*abaI*(F)	ATGAATATTATTGCTGGA	Surface motility
	abaI(R)	CTACACATCAATCAAGCA	
17	16S rRNA (F)	ACTCCTACGGGAGGCAGCAG	Housekeeping gene
	16S rRNA (R)	ATTACCGCGGCTGCTGG	
18	*erg11*(F)	GAAAGAGAACCATTACCAGG	Azole resistance
	erg11(R)	AGGAATCGACGGATCAC	
19	*cdr*(F)	TGGTGCCATGACTCCTGCTA	Efflux gene
	cdr(R)	CCATCGAGACCAACCCAACA	
20	*efg* (F)	CCAGGGTGGTGCTGCTAATG	Biofilm formation
	efg (R)	GGGTGAAGGGTGAACTGAACC	
21	*hgc* (F)	GCTTCCTGCACCTCATCAAT	Morphogenesis and biofilm formation
	hgc (R)	AGCACGAGAACCAGCGATAC	
22	ITS (F)	TCCGTAGGTGAACCTGCGG	Housekeeping gene

	ITS (R)	TCCTCCGCTTATTGATATGC	
